# Expression of Tryptophan Metabolism Enzymes in Patients with Diffuse Large B‐cell Lymphoma and NK/T‐cell Lymphoma

**DOI:** 10.1002/cam4.5903

**Published:** 2023-05-06

**Authors:** Dan Guo, Yuming Wang, Xunyao Wu, Yike Gao, Anqi Wang, Zixin Zhang, Kun Zhao, Xiaoxi Wang, Meiyu Liu, Yaran Zhang, Mei Li, Rui Chen, Jian Sun, Yan Zhang

**Affiliations:** ^1^ Department of Medical Research Center, Peking Union Medical College Hospital Chinese Academy of Medical Sciences and Peking Union Medical College Beijing China; ^2^ Clinical Biobank, Peking Union Medical College Hospital Chinese Academy of Medical Sciences and Peking Union Medical College Beijing China; ^3^ Department of Pathology, Peking Union Medical College Hospital Chinese Academy of Medical Sciences and Peking Union Medical College Beijing China; ^4^ Department of Hematology, Peking Union Medical College Hospital Chinese Academy of Medical Sciences and Peking Union Medical College Beijing China

**Keywords:** diffuse large B‐cell lymphoma, immunohistochemistry, natural killer/T‐cell lymphoma, programmed death‐ligand 1, tryptophan metabolism

## Abstract

**Background:**

Metabolites of tryptophan (Trp) metabolism in the tumor microenvironment play crucial immunosuppressive roles in various cancers. However, the role of Trp metabolism in diffuse large B‐cell lymphoma (DLBCL) or natural killer/T‐cell lymphoma (NK/TCL) remains unelucidated.

**Methods:**

We investigated the potential role of Trp metabolism in a cohort of 43 patients with DLBCL and 23 with NK/TCL. We constructed tissue microarrays and performed in situ staining of Trp‐catabolizing enzymes and PD‐L1 using immunohistochemistry (IHC).

**Results:**

We observed 14.0% positive staining of IDO1 in DCBCL and 60.9% in NK/TCL; 55.8% of IDO2 in DCBCL and 95.7% in NK/TCL; 79.1% of TDO2 in DCBCL and 43.5% in NK/TCL; 29.7% of IL4I1 in DCBCL and 39.1% in NK/TCL. However, IDO1, IDO2, TDO2, and IL4I1 positivity did not significantly differ between PD‐L1+ and PD‐L1− biopsy tissue samples of NK/TCL; nonetheless, a positive correlation of IDO1 (*r* = 0.87, *p* < 0.001), IDO2 (*r* = 0.70, *p* < 0.001), TDO2 (*r* = 0.63, *p* < 0.001), and IL4I1 (*r* = 0.53, *p* < 0.05) with PD‐L1 expression was observed in the TCGA‐DLBCL dataset. Finally, immunohistochemical (IHC) analysis revealed the lack of superior prognostic effect with higher expression of Trp enzymes in DLBCL and NK/TCL. Furthermore, IDO1, IDO2, TDO2, and IL4I1 expression, as well as survival rates, did not significantly differ across all groups in the TCGA‐DLBCL cohort.

**Conclusion:**

Collectively, our findings provide novel insights into the enzymes involved in Trp metabolism in DLBCL and NK/TCL and their association with PD‐L1 expression, which offers potential strategies to combine Trp‐metabolism enzyme inhibitors with anti‐PD‐L1 or other immunotherapeutic strategies in clinical DLBCL or NK/TCL treatment.

## Graphical abstract

The degradation of Tryptophan to Kynurenine is catabolized by tryptophan enzymes, including IDO1, IDO2, TDO2, and IL4I1, which up‐regulate PD‐L1 expression and promote tumor suppressive microenvironment in various cancers. In the present study, we constructed tissue microarrays and performed in situ staining of Trp‐catabolizing enzymes and PD‐L1 using immunohistochemistry in the diffuse large B‐cell lymphoma (DLBCL) or natural killer/T‐cell lymphoma (NK/TCL). We provide potential strategies for Trp metabolism enzyme inhibitors in combination with anti‐PD‐L1 or other immunotherapeutic strategies in clinical DLBCL or NK/TCL treatment.

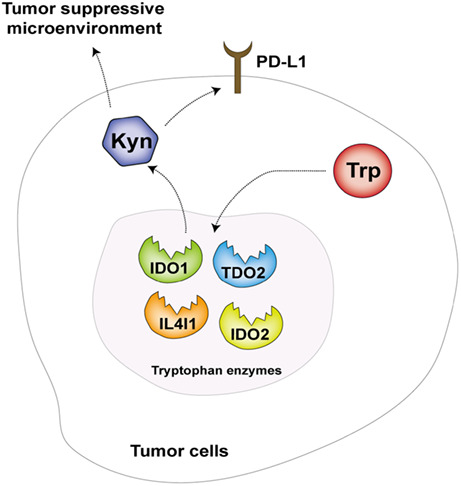

## 1 INTRODUCTION

Non‐Hodgkin lymphoma, which comprises lymphoproliferative disorders originating in B cell, T cell, or natural killer (NK) cells, is the leading type of cancer worldwide.^1^ Diffuse large B‐cell lymphoma (DLBCL) is the most common type of non‐Hodgkin lymphoma in adults worldwide.^2^ Only 40% of DLBCL cases respond well to current therapy, with a 5‐year overall survival rate of 60%–70%.^2^ Natural killer/T‐cell lymphoma (NK/TCL), the most common neoplasm of mature T‐cells and NK cells in Asia,^3^ is a rare but highly aggressive lymphoma. Currently, no standard treatment strategy has been established, and the outcome of NK/TCL remains suboptimal.^4^

Immunotherapy, such as anti‐programmed death‐1 (anti‐PD‐1) or anti‐programmed death‐ligand 1 (anti‐PD‐L1) therapy, which blocks immune checkpoints, exhibits considerable therapeutic effects on specific lymphoma subtypes, including classical Hodgkin lymphoma (CHL). However, anti‐PD‐1/PD‐L1 therapy provides suboptimal responses in DLBCL and NK/TCL.^5^

A possible barrier to effective immunotherapy is the hostile immunosuppressive tumor microenvironment (TME) induced by tumor‐mediated amino acid metabolism.^6^ Abnormal metabolic reprogramming in the TME is implicated in the regulation of antitumor immune response.^7^

The immunosuppressive role of tryptophan (Trp) metabolism has been involved in various cancers. Trp is an essential amino acid with three major downstream pathways: serotonin, indoleacetic, and kynurenine (Kyn).^8^ The Kyn pathway plays an immunosuppressive role in various cancer cell types.^9^

The degradation of Trp to Kyn is catabolized by indoleamine‐2,3‐dioxygenase 1 and 2 (IDO1/2) and tryptophan‐2,3‐dioxygenase (TDO2).^10^ IDO1 is the most widely studied Trp‐catabolizing enzyme and can be detected in tumor, immune, and endothelial cells in various tissues, including the intestine, pancreas, kidney, and central nervous system.^11^, ^12^, ^13^, ^14^ IDO2 is a more narrowly expressed protein than IDO1 and is confined mainly to antigen‐presenting immune cells in the liver, kidney, brain, and placenta.^15^ IDO2 is overexpressed in non‐small cell lung cancer,^16^ pancreatic cancer,^17^, ^18^ colon cancer, gastric cancer, and renal tumors.^19^ Meanwhile, TDO2 is expressed only in the liver and is involved in Trp metabolism under physiological conditions. Overexpression of TDO2 in tumors, such as breast cancer, hepatocellular carcinoma, and bladder cancer, is correlated with poor prognosis and therapy resistance.^20^, ^21^, ^22^, ^23^ Recently, increased interleukin‐4‐induced‐1 (IL4I1) expression has been reported to catabolize Trp into Kyn and has been implicated in immune regulatory functions in cancer and metastasis.^24^

However, the role of Trp metabolism in DLBCL or NK/TCL remains unclear. Therefore, in the present study, we aimed to investigate the in situ expression of Trp metabolism enzymes, including IDO1, IDO2, TDO2, and IL4I1 in DLBCL and NK/TCL, using immunohistochemistry (IHC) in tissue microarrays.

## 2 MATERIALS AND METHODS

### 2.1 Patients and tumor specimens

We included 66 patients diagnosed with DLBCL or NK/TCL in our study. All patients underwent surgical treatment at Peking Union Medical College Hospital (Beijing, China) between 2010 and 2016. The patients were re‐diagnosed by two experienced pathologists blinded to the first diagnosis based on the WHO guidelines introduced in 2017. Clinical data were collected, including the patient's age, gender, clinical stage, pathological diagnosis, and overall survival.

Formalin‐fixed, paraffin‐embedded tissues were used to generate a tissue microarray (TMA) for immunohistochemical (IHC) staining.

This study was approved by the Institutional Review Board of Peking Union Medical College Hospital (I‐22PJ112). Informed consent was obtained from all the patients.

### 2.2 Immunohistochemical staining

IHC staining was performed on 4‐μm paraffin sections using the DAKO Autostainer Link 48. Tissue epitopes were repaired using an automated water bath heating process with a Dako PT Link (Dako). The sections were incubated in TRIS‐EDTA retrieval solution (10 mM Tris, 1 mM EDTA, pH 9.0) or citric acid buffer (10 mM, pH 6.0) at 98°C for 20 min. They were subsequently incubated for 20 min with primary antibodies, followed by anti‐rabbit immunoperoxidase polymer (Envision FLEX/HRP) for 20 min. Primary antibodies included IDO1 (EPR20374, Abcam, ab211017, dilution 1:4000), IDO2 (8,322,548, Porteintech, 25,053‐1‐AP, dilution 1:100), TDO2 (4,071,714, Proteintech, 15,880‐1‐AP, dilution 1:100), IL4I1 (EPR22070, Abcam, ab222102, dilution 1:2000), and PD‐L1 (28–8, Dako). The colorimetric reaction was developed using a DAB substrate‐chromogen solution for 10 min. Finally, the sections were counterstained with hematoxylin.

PD‐L1 was expressed on the membranes. Diffuse cytoplasmic staining was observed for IDO1, IDO2, and TDO2. IL4I1 was scattered throughout the cytoplasm as granular. The IHC controls are listed below.

For PD‐L1, lung squamous cell carcinoma with PD‐L1 positivity and the NCL‐H226 and MCF‐7 cell lines were used as positive and negative controls, respectively. Additionally, the tissues of endometrial carcinoma, normal liver, hepatocellular carcinoma, and DLBCL tissues were used as positive controls for IDO1, IDO2, TDO2, and IL4I1 antibodies, respectively.

Composite scores for IDO1, IDO2, TDO2, and IL4I1 were evaluated by two experienced pathologists based on both the intensity and proportion of positive cells. The intensity score was scored on a scale of 0–3 (0: negative; 1: weak; 2: moderate; 3: intense staining), and the percentage of positive cells was scored on a scale of 1–4 (1: 0%–24%; 2: 25%–49%; 3: 50%–74%; 4: 75%–100%). Sections with composite scores (product of intensity score and percentage) ≥4 points were considered positive. PD‐L1 expression was scored and classified independently by two experienced individuals, as in a previous study.^25^

### 2.3 Bioinformatic analysis

Datasets of 26 patients diagnosed with DLBCL were downloaded from The Cancer Genome Atlas (TCGA) and selected for further analysis. The details of the clinical information are presented in Table S1.

### 2.4 Statistical analysis

Statistical analyses were performed using SPSS with nonparametric statistics for IHC of IDO1, IDO2, TDO2, IL4I1, and PD‐L1 expression and clinical characteristics. Pearson's coefficient was calculated to evaluate the relationship between the expression of PD‐L1, IDO1, IDO2, TDO2, and IL4I1 and clinical characteristics. Kaplan–Meier (K–M) analysis was used to explore the prognostic value of IDO1, IDO2, TDO2, and IL4I1. The data were analyzed using SPSS version 24.0 for Windows (SPSS Inc.). Statistical significance was set at *p* < 0.05.

## 3 RESULTS

### 3.1 Clinical characterization

Overall, 43 DLBCL and 23 NK/TCL biopsy tissue samples were analyzed in the present study. The clinical features and characteristics of the patients are summarized in Table S2. The average age of patients with DLBCL and NK/TCL were 56 and 43 years, respectively; patients with DLBC and NK/TCL aged <60 years comprise 46.5% and 26.1% of the included patients, respectively.

### 3.2 Immunohistochemical expression

**FIGURE 1:**
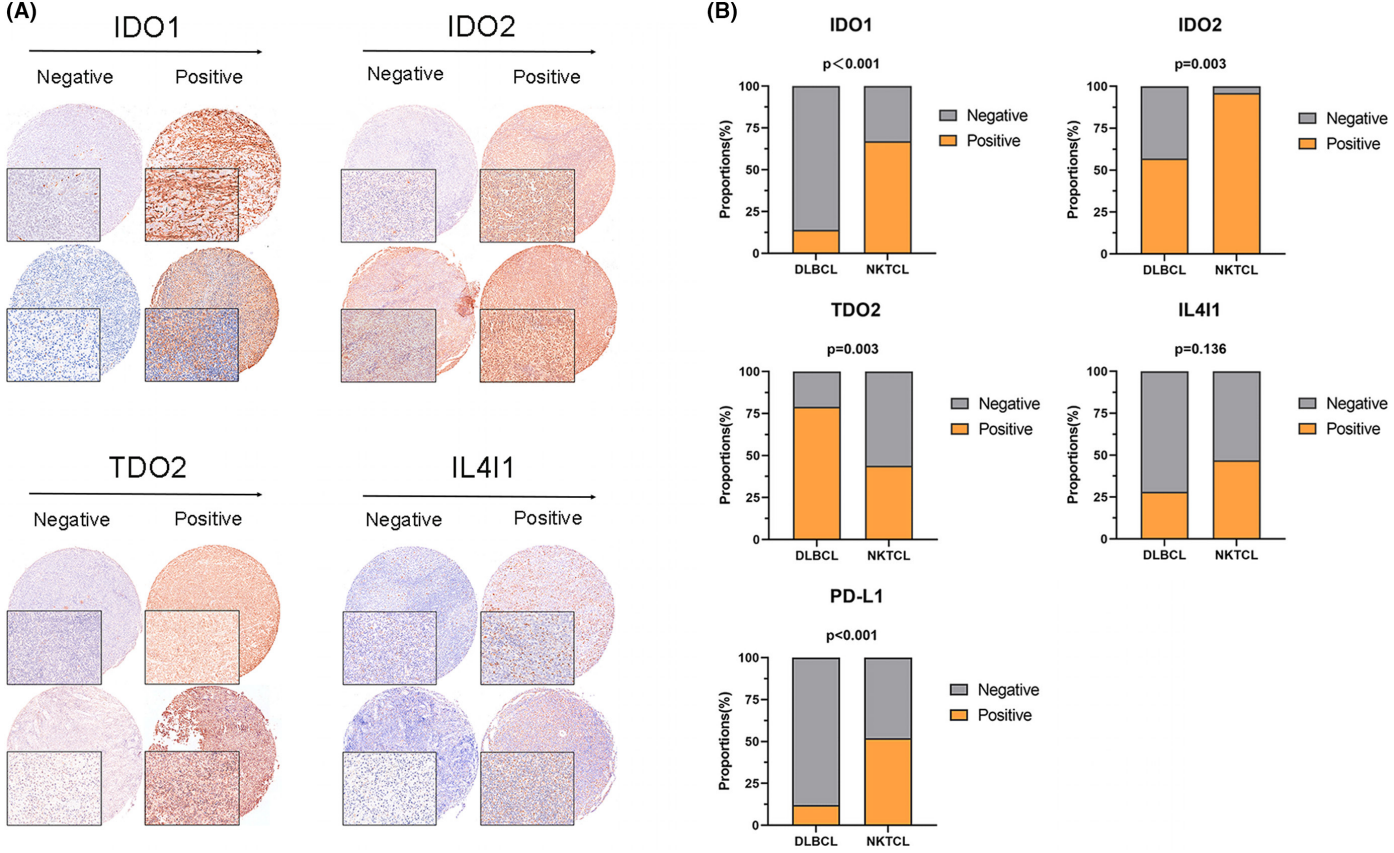
Expression of tryptophan metabolizing enzymes using immunohistochemical staining in DLBCL and NK/TCL. (A) Representative images of IHC staining of IDO1, IDO2, TDO2, and IL4I1 in DLBCL and NK/TCL. (B) Analysis of the proportions of IDO1, IDO2, TDO2, and IL4I1 (negative, positive) in DLBCL and NK/TCL. DLBCL, diffuse large B‐cell lymphoma; IHC, immunohistochemical; PD‐L1, programmed death‐ligand 1; NK/TCL, natural killer/T‐cell lymphoma.

We evaluated the IHC staining of Trp‐catabolizing enzymes, including IDO1, IDO2, TDO2, and IL4I1, in tumor cells (Figure 1). Representative examples of different expression levels of IDO1, IDO2, TDO2, and IL4I1 in DLBCL and NK/TCL are displayed in Figure 1A according to previous studies.^25^, ^26^, ^27^, ^28^, ^29^ As shown in Tables 1 and 2, positive staining for IDO1, IDO2, TDO2, and IL4I1 was observed in 6 (14.0%), 24 (55.8%), 34 (79.1%), and 12 (27.9%) of 43 DLBCL cases and in 14 (60.9%), 22 (95.7%), 10 (43.5%), and 9 (39.1%) of 23 NK/TCL cases, respectively. The positive ratio of PD‐L1 in DLBCL was 11.6% (5/43), whereas that in NK/TCL was 52.4% (11/21).

**TABLE 1:** Relationship between TDO2/IDO1/IDO2/IL4I1/PD‐L1 expression and clinical characteristics in DLBCL (*n* = 43).

Clinicopathologic parameters	IDO1 (*n* = 42)			IDO2 (*n* = 42)			TDO2 (*n* = 43)			IL4I1 (*n* = 43)			PD‐L1 (*n* = 43)		
	Negative (%)	Positive (%)	*p*	Negative (%)	Positive (%)	*p*	Negative (%)	Positive (%)	*p*	Negative (%)	Positive (%)	*p*	Negative (%)	Positive (%)	*p*
Age			1.000			0.327			1.000			1.000			0.910
≥60	16 (44.4)	3 (50.0)		7 (38.9)	13 (54.2)		4 (44.4)	16 (47.1)		14 (45.2)	6 (50.0)		17 (44.7)	2 (40.0)	
<60	20 (55.6)	3 (50.0)		11 (61.1)	11 (45.8)		5 (55.6)	18 (52.9)		17 (54.8)	6 (50.0)		21 (55.3)	3 (60.0)	
Gender			1.000			0.789			0.813			0.692			0.868
Female	20 (55.6)	3 (50.0)		9 (50.0)	13 (54.2)		4 (44.4)	19 (55.9)		16 (51.6)	7 (58.3)		21 (55.3)	2 (40.0)	
Male	16 (44.4)	3 (50.0)		9 (50.0)	11 (45.8)		5 (55.6)	15 (44.1)		15 (48.4)	5 (41.7)		17 (44.7)	3 (60.0)	
Advanced Lugano stage^a^			0.712			1.000			1.000			1.000			1.000
I/II	6 (20.7)	2 (40.0)		4 (25.0)	4 (22.2)		2 (25.0)	6 (22.2)		6 (24.0)	2 (20.0)		7 (23.3)	1 (20.0)	
III/IV	23 (79.3)	3 (60.0)		12 (75.0)	14 (77.8)		6 (75.0)	21 (77.8)		19 (76.0)	8 (80.0)		23 (76.7)	4 (80.0)	
LDH^a^			0.733			0.373			0.861			0.877			0.510
Normal (120–250 U/L)	15 (51.7)	3 (75.0)		9 (60.0)	8 (44.4)		3 (42.9)	15 (55.6)		12 (50.0)	6 (60.0)		17 (56.7)	1 (25.0)	
Elevated (>250 U/L)	14 (48.3)	1 (25.0)		6 (40.0)	10 (55.6)		4 (57.1)	12 (44.4)		12 (50.0)	4 (40.0)		13 (43.3)	3 (75.0)	

^a^ Some cases were not taken into calculation due to loss of clinical data.

**TABLE 2:** Relationship between TDO2/IDO1/IDO2/IL4I1 expression and clinical characteristics in NK/TCL (*n* = 23).

Clinicopathologic parameters	IDO1 (*n* = 21)			IDO2 (*n* = 23)			TDO2 (*n* = 23)			IL4I1 (*n* = 19)			PD‐L1 (*n* = 21)		
	Negative (%)	Positive (%)	*p*	Negative (%)	Positive (%)	*p*	Negative (%)	Positive (%)	*p*	Negative (%)	Positive (%)	*p*	Negative (%)	Positive (%)	*p*
Age			1.000			1.000			0.052			1.000			0.149
≥60	2 (28.6)	3 (21.4)		0 (0.0)	6(27.3)		1 (7.7)	5 (50.0)		3 (30.0)	2 (22.2)		1 (10.0)	5 (45.5)	
<60	5 (71.4)	11 (78.6)		1 (100.0)	16 (72.7)		12 (92.3)	5 (50.0)		7 (70.0)	7 (77.8)		9 (90.0)	6 (54.5)	
Gender			1.000			1.000			0.104			0.582			0.311
Female	1 (14.3)	3 (21.4)		0 (0.0)	4 (18.2)		4 (30.8)	0 (0.0)		1 (10.0)	2 (22.2)		3 (30.0)	1 (9.1)	
Male	6 (85.7)	11 (78.6)		1 (100.0)	18 (81.8)		9 (69.2)	10 (100.0)		9 (90.0)	7 (77.8)		7 (70.0)	10 (90.9)	
Advanced Lugano stage^a^			1.000			0.438			1.000			1.000			1.000
I/II	2 (50.0)	5 (50.0)		0 (0.0)	9 (60.0)		5 (55.6)	4 (57.1)		5 (62.5)	3 (50.00)		4 (57.1)	5 (55.6)	
III/IV	2 (50.0)	5 (50.0)		1 (100.0)	6 (40.0)		4 (44.4)	3 (42.9)		3 (37.5)	3 (50.00)		3 (42.9)	4 (44.4)	
LDH^a^			1.000			1.000			0.315			0.301			0.358
Normal (120–250 U/L)	2 (50.0)	4 (40.0)		0 (0.0)	7 (46.7)		5 (62.5)	2 (25.0)		5 (55.6)	1 (20.0)		2 (28.6)	5 (55.6)	
Elevated (>250 U/L)	2 (50.0)	6 (60.0)		1 (100.0)	8 (53.3)		3 (37.5)	6 (75.0)		4 (44.4)	4 (80.0)		5 (71.4)	4 (44.4)	

^a^ Some cases were not taken into calculation due to loss of clinical data.

We then compared the IHC results and clinical characteristics between DLBCL and NK/TCL. A significant correlation was not observed between IDO1, IDO2, TDO2, IL4I1, and PD‐L1 expression and clinical characteristics in DLBCL or NK lymphoma; however, PD‐L1 (*p* < 0.001), IDO1 (*p* < 0.001), and IDO2 (*p* = 0.003) were expressed at a higher proportion in NK/TCL than in DLBCL, whereas TDO2 often exhibited higher expression levels in DLBCL than in NK/TCL (*p* = 0.003) (Figure 1B).

### 3.3 Correlation of IDO1/IDO2/TDO2/IL4I1 expression with that of PD‐L1

**FIGURE 2:**
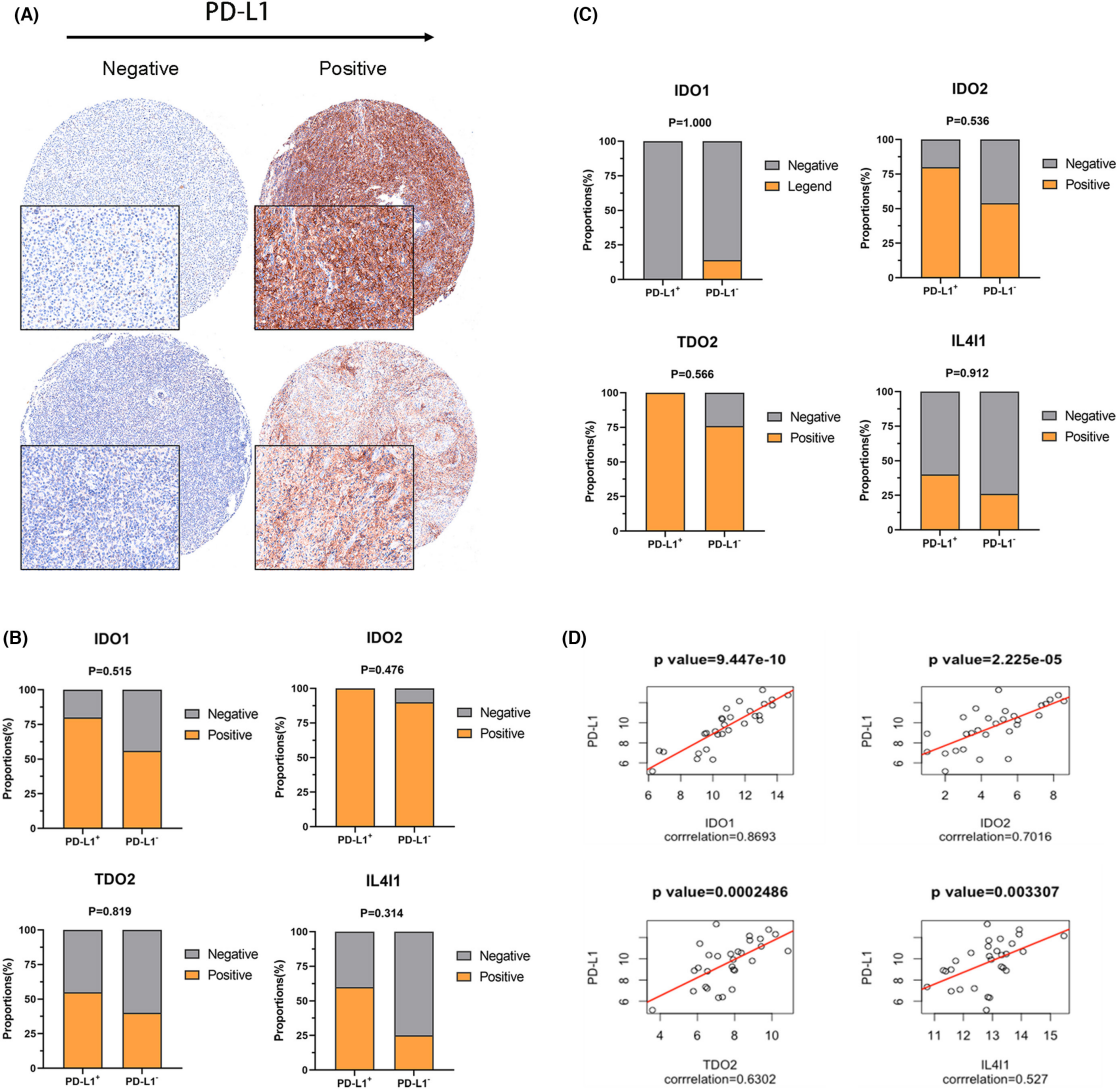
Correlation of tryptophan metabolizing enzymes with PD‐L1 in DLBCL and NK/TCL (A) Representative images of IHC staining for PD‐L1 in DLBCL and NK/TCL. (B) Analysis of the proportions of IDO1, IDO2, TDO2, and IL4I1 (Negative, Positive) in PD‐L1 positive and PD‐L1 negative samples in NK/TCL. (C) Analysis of the proportions of IDO1, IDO2, TDO2, and IL4I1 (Negative, Positive) in PD‐L1 positive and PD‐L1 negative samples in DLBCL samples. (D) Correlation analysis of IDO1, IDO2, TDO2, and IL4I1 with PD‐L1 in the TCGA cohort of DLBCL.DLBCL, diffuse large B‐cell lymphoma; IHC, immunohistochemical; PD‐L1, Programmed death‐ligand 1; NK/TCL, Natural killer/T‐cell lymphoma.

Finally, we explored the relationship between IDO1, IDO2, TDO2, and IL4I1 and PD‐L1 expression. Representative images of PD‐L1 IHC staining are displayed in Figure 2A. We did not observe a statistical difference in IDO1, IDO2, TDO2, and IL4I1 expression between PD‐L1+ and PD‐L1− samples in NK/TCL (Figure 2B) or DLBCL (Figure 2C) using IHC analysis. Furthermore, we performed correlation studies in the TCGA database and observed a positive correlation between IDO1 (*r* = 0.87, *p* < 0.001), IDO2 (*r* = 0.70, *p* < 0.001), TDO2 (*r* = 0.63, *p* < 0.001), and IL4I1 (*r* = 0.53, *p* < 0.01) with PD‐L1 expression in DLBCL (Figure 2D).

### 3.4 Clinicopathological and prognostic impact of the expression of Trp‐metabolizing enzymes in DLBCL and NK/TCL

We observed no significant correlations between IDO1, IDO2, TDO2, and IL4I1 expression and clinical characteristics of patients with DLBCL or NK/TCL (Tables 1 and 2). Furthermore, PD‐L1 expression was not associated with any clinicopathological features of DLBCL or NK/TCL (Tables 1 and 2).

**FIGURE 3:**
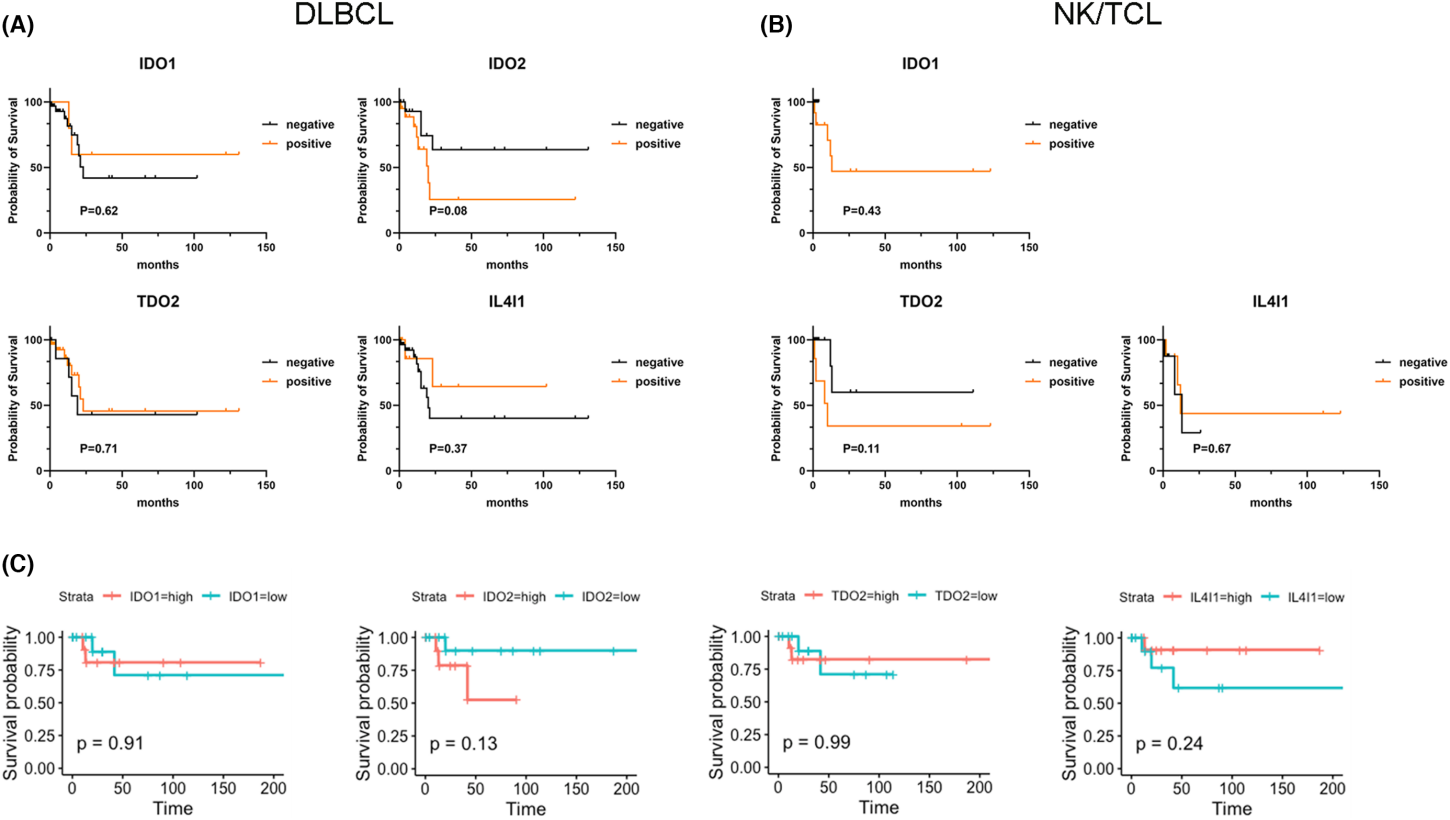
Survival analysis of tryptophan‐metabolizing enzymes in DLBCL and NK/TCL. Kaplan–Meier survival curves of (A) IDO1, IDO2, TDO2, and IL4I1 in DLBCL; (B) IDO1, TDO2, and IL4I1 in NK/TCL; (C) IDO1, IDO2, TDO2, and IL4I1 in TCGA cohort of DLBCL patients. DLBCL, diffuse large B‐cell lymphoma; NK/TCL, natural killer/T‐cell lymphoma.

Overall survival analysis indicated the lack of significant differences in the protein expression of IDO1, IDO2, TDO2, and IL4I1 in DLBCL and NK/TCL groups using IHC analysis (Figure 3A,B). Since only one case revealed negative IDO2 protein expression in tumor cells, the survival analysis on IDO2 protein expression in NK/TCL could not be performed.

Furthermore, TCGA validation of correlations in DLBCL patients confirmed the lack of statistical difference across all the compared groups between IDO1, IDO2, TDO2, and IL4I1 expression and survival rate (Figure 3C), which was consistent with our results.

## 4 DISCUSSION

Trp metabolism yields metabolites that activate the aryl hydrocarbon receptor, which enhances the aggressiveness of the tumor and suppresses antitumor immunity.^30^ In the present study, we analyzed the expression of Trp enzymes, including IDO1, IDO2, TDO2, and IL4I1, in patients with DLBCL and NK/TCL. We demonstrated that IDO2, TDO2, and IL4I1 are preferentially expressed in DLBCL patients, whereas all four enzymes are expressed in approximately half of NK/TCL patients. Using datasets from TCGA, we further observed that the expression of IDO1, IDO2, TDO2, and IL4I1 was positively correlated with PD‐L1 in DLBCL patients. Our study is the first comprehensive, in situ assessment of Trp‐metabolizing enzyme expression in DLBCL and NK/TCL tumor cells. The coexistence of IDO1, IDO2, TDO2, and IL4I1 highlighted the potential biological significance of Trp metabolism in both DLBCL and NK/TCL. Therefore, even though the better prognostic effect of high Trp enzyme expression was not observed in DLBCL and NK/TCL by IHC analysis and in the TCGA cohort, we inferred that high Trp metabolism in DLBCL and NK/TCL in tumor cells might still play an important role in inducing immunosuppressive microenvironment and targeting Trp enzymes might be a key drug to improve the efficacy of immunotherapy.

Notably, the Trp metabolic pathway differs among the different lymphoma subtypes. We discovered that most DLBCL cells expressed TDO2 but lacked the expression of IDO1 and IDO2, whereas the reverse was observed in NK/TCL. IDO1, IDO2, and TDO2 contribute to the noteworthy local catabolism of Trp, and these enzymes, along with PD‐L1, play a crucial role in tumor immune escape. Despite sharing the same catabolic function, IDO1 has a wider distribution in different tissues than that of IDO2 and TDO2 under normal conditions.^31^ Owing to its frequent expression in human tumors (approximately 60%) and the major degradation of *L*‐Trp, IDO1 is considered a promising immunotherapeutic target.

To date, no Trp‐metabolizing enzyme has been combined with PD‐1/PD‐L1 immunotherapy in DLBCL and NK/TCL. A previous phase III clinical trial in advanced melanoma examined the clinical benefit of combining an IDO1 inhibitor (epacadostat) with a PD‐1 inhibitor (pembrolizumab); the results indicated that patients failed to benefit significantly from the treatment.^32^ Another phase I study of the IDO1 inhibitor (navoximod) in combination with a PD‐L1 inhibitor (atezolizumab) in advanced solid tumors did not confer significant benefit.^33^ In our study, the expression of IDO1 and IDO2 differed between DLBCL and NK/TCL, indicating that IDO1/2‐targeted therapy might have a better effect in NK/TCL than in DLBCL and that TDO2 may play a more vital role in DLBCL than in NK/TCL. As the failure of IDO1 inhibitors in clinical trials might be a cause of the compensation effect offered by IDO2 or TDO2, IDO1/IDO2 or IDO1/TDO2 dual‐target inhibitors are a better choice for target therapy.^31^

A considerable proportion expressed Trp‐catabolizing enzymes in either PD‐L1 positive or negative cases. Targeting Trp metabolism may be a promising strategy for resistance to inhibition of the PD‐1/PD‐L1 axis. Clinical studies in the early phase have demonstrated encouraging outcomes with the addition of IDO1 inhibitors to PD‐1 or PD‐L1 in advanced solid tumors.^33^, ^34^, ^35^

In our tissue microarray analysis, we observed 27.9% and 39.1% IL4I1 expression in DLBCL and NK/TCL, respectively, which were higher than those in previous studies. Christiane et al. reported that IL4I1 is activated in primary mediastinal large B‐cell lymphoma.^36^ They further analyzed several lymphoma subtypes^37^ and observed no cases of IL4I1‐positive tumor cells in NK/TCL, and 89% of primary mediastinal large B‐cell lymphomas were positive for IL4I1. However, most non‐mediastinal DLBCL cases are IL4I1‐negative, with only a minority of cases (17%) exhibiting occasional IL4I1‐positive tumor cells.^37^ Moreover, high IL4I1 expression was associated with the absence of bone marrow involvement and a better outcome. Choueiry et al. integrated metabolomics and gene expression profiling and elucidated *IL4I1* as a modulator of ibrutinib resistance in activated B‐cell (ABC) DLBCL.^38^ Therefore, further investigation is required to confirm the roles and explore the potential mechanisms of IL4I1 in DLBCL and NK/TCL in future studies.

In our present study, PD‐L1 expression was significantly higher in NK/TCL than in DLBCL, which is in accordance with a previous study.^39^ Panjwani et al. measured the expression of PD‐L1 protein using IHC and observed that PD‐L1 staining was detected in 53% of primary mediastinal large B‐cell lymphomas, 39% of extranodal NK/T‐cell lymphomas, and 10% of DLBCL,^40^ which was consistent with our study. Jo et al. reported up to 79.7% PD‐L1 expression rate in extranodal NK/TCL.^41^ However, PD‐L1 mRNA expression in normal human B cells was lower than that in CD4^+^ T, CD8^+^ T, and NK cells.^42^ Furthermore, the poor prognosis and chemotherapy resistance of NK/TCL may be attributed to immune escape mediated by PD‐L1 expression.^39^ Upregulated PD‐L1 expression is reportedly associated with the rapid progression and poor prognosis of DLBCL.^43^, ^44^ In our study, PD‐L1 expression in DLBCL by 11.6% was significantly lower than that in previous reports,^39^, ^45^ that might be because our staining assessment has been relatively more rigorous (immunohistoscore >10).

Even among the DLBCL and NK/TCL patients in the present study, the expression of Trp‐metabolizing enzymes was not associated with prognosis. In the context of oncology therapy, differential expression of these enzymes may be a key drug target for improving the efficacy of immunotherapy. Our study may be beneficial for future relevant clinical translational studies and therapeutic applications.

Our study had several limitations. The main limitation is the relatively small sample size, which might have led to bias in the conclusion. Although the correlation analysis of IDO1, IDO2, TDO2, and IL4I1 with PD‐L1 in the TCGA cohort was quite different from the IHC results in our present study, several reasons might explain the difference: (1) The expressions of IDO1, IDO2, TDO2, and IL4I1 in the TCGA cohort are from the whole tissues, which includes intra‐tumor cells, non‐tumor epithelial cells, and immune cells. Our study only focuses on their expressions in intra‐tumor cells, highlighting their potential roles in tumorigenesis and development. (2) The expressions of IDO1, IDO2, TDO2, and IL4I1 are displayed in mRNA levels with a continuous numerical value in the TCGA cohort. While in our present study, they are displayed in protein levels and scored with both intensity and the percentage of positive cells on a scale of 0–3 and 1–4, respectively. (3) The TCGA cohort included 9 Asians (34.6%), 1 Black or African American (3.8%), and 16 Whites (61.5%)—races that differed from our study. Moreover, functional assays validating the role of Trp metabolism in DLBCL and NK/TCL were not performed. Therefore, we intend to expand our sample size and perform functional experiments to further explore the mechanisms by which Trp metabolism regulates disease progression in DLBCL and NK/TCL.

## 5 CONCLUSION

Our study is the first to explore the enzymes of Trp metabolism in DLBCL and NK/TCL and their association with protein expression of PD‐L1 in the TME. As both Trp metabolites and PD‐L1 exhibit critical immunosuppressive roles in the TME, we provide potential strategies for Trp metabolism enzyme inhibitors in combination with anti‐PD‐L1 or other immunotherapeutic strategies in clinical DLBCL or NK/TCL treatment.

## AUTHOR CONTRIBUTIONS

**Dan Guo:** Conceptualization (equal); data curation (equal); funding acquisition (equal); project administration (equal); resources (equal); supervision (equal). **Yuming Wang:** Formal analysis (equal); methodology (equal); validation (equal); visualization (equal); writing – original draft (equal); writing – review and editing (supporting). **Xunyao Wu:** Conceptualization (equal); formal analysis (equal); methodology (supporting); software (equal); validation (equal); writing – original draft (equal). **Yike Gao:** Data curation (equal); formal analysis (supporting); visualization (equal); writing – review and editing (supporting). **Anqi Wang:** Funding acquisition (equal); resources (equal); visualization (equal). **Zixin Zhang:** Methodology (equal); visualization (equal). **Kun Zhao:** Data curation (supporting); methodology (equal); validation (supporting). **Xiaoxi Wang:** Data curation (equal); resources (equal); visualization (supporting). **Meiyu Liu:** Data curation (supporting); funding acquisition (equal); resources (equal). **Yaran Zhang:** Investigation (equal); project administration (supporting); software (supporting). **Mei Li:** Investigation (equal); methodology (equal); resources (supporting). **Rui Chen:** Data curation (equal); resources (equal). **Jian Sun:** Conceptualization (equal); project administration (lead); resources (supporting); supervision (lead); writing – review and editing (equal). **Yan Zhang:** Conceptualization (equal); methodology (supporting); project administration (equal); resources (equal); writing – review and editing (supporting).

## FUNDING INFORMATION

This study was supported by the National High Level Hospital Clinical Research Funding (LY23F00410430), CAMS Innovation Fund for Medical Sciences (CIFMS) (2021‐I2M‐1‐053), and National High Level Hospital Clinical Research Funding (2022‐PUMCH‐D‐002).

## CONFLICT OF INTEREST STATEMENT

The authors declare no conflicts of interest.

## ETHICS STATEMENT

This study was performed in accordance with the Declaration of Helsinki and approved by the Ethics Committee Review Board of Peking Union Medical College Hospital, China (ethics number: I‐22PJ112).

## CONSENT STATEMENT

Each patient/control provided written informed consent.

## Supporting information


Table S1.

Table S2.

## Data Availability

Data sharing is not applicable to this article as no new data were created or analyzed in this study.
