# Supplementary material for: Expression of Tryptophan Metabolism Enzymes in Patients with Diffuse Large B‐cell Lymphoma and NK/T‐cell Lymphoma
Source: Cancer Med. 2023 May 6;12(11):12139–48. doi: 10.1002/cam4.5903 (PMC10278463; doi:10.1002/cam4.5903)
Supplement: Supplementary file 1 — Table S1. [file CAM4-12-12139-s001.pdf]

## Table S1. Details of clinical information downloaded from TCGA datasets

| | histological_type | gender | days_to_birth | race_list | days_to_initial_pathologic_diagnosis | age_at_initial_pathologic_diagnosis | year_of_initial_pathologic_diagnosis | stage_event | vital_status |
|---|---|---|---|---|---|---|---|---|---|
| 1 | Diffuse large B-cell lymphoma (DLBCL) NOS (any anatomic site nodal or extranodal) | MALE | -24569 | WHITE | 0 | 67 | 1996 | 4thStage IIINONO | Alive |
| 2 | Diffuse large B-cell lymphoma (DLBCL) NOS (any anatomic site nodal or extranodal) | FEMALE | -22454 | ASIAN | 0 | 61 | 2013 | 7thStage INONO | Alive |
| 3 | Diffuse large B-cell lymphoma (DLBCL) NOS (any anatomic site nodal or extranodal) | MALE | -24590 | WHITE | 0 | 67 | 2012 | 7thStage IVYESYES | Alive |
| 4 | Diffuse large B-cell lymphoma (DLBCL) NOS (any anatomic site nodal or extranodal) | FEMALE | -24405 | WHITE | 0 | 66 | 2009 | Stage IVNOYES | Alive |
| 5 | Diffuse large B-cell lymphoma (DLBCL) NOS (any anatomic site nodal or extranodal) | FEMALE | -23641 | WHITE | 0 | 64 | 2013 | 7thStage IIINONO | Alive |
| 6 | Diffuse large B-cell lymphoma (DLBCL) NOS (any anatomic site nodal or extranodal) | FEMALE | -15750 | BLACK OR AFRICAN AMERICAN | 0 | 43 | 2006 | 6thStage IVNOYES | Dead |
| 7 | Diffuse large B-cell lymphoma (DLBCL) NOS (any anatomic site nodal or extranodal) | MALE | -21272 | WHITE | 0 | 58 | 1987 | YESYES | Dead |
| 8 | Diffuse large B-cell lymphoma (DLBCL) NOS (any anatomic site nodal or extranodal) | MALE | -24661 | WHITE | 0 | 67 | 2002 | 5thStage IIINONO | Alive |
| 9 | Diffuse large B-cell lymphoma (DLBCL) NOS (any anatomic site nodal or extranodal) | MALE | -27025 | ASIAN | 0 | 73 | 2013 | 7thNONO | Alive |
| 10 | Diffuse large B-cell lymphoma (DLBCL) NOS (any anatomic site nodal or extranodal) | FEMALE | -10075 | WHITE | 0 | 27 | 2011 | 7thStage IINONO | Dead |
| 11 | Diffuse large B-cell lymphoma (DLBCL) NOS (any anatomic site nodal or extranodal) | MALE | -13317 | WHITE | 0 | 36 | 2005 | Stage IINONO | Alive |
| 12 | Diffuse large B-cell lymphoma (DLBCL) NOS (any anatomic site nodal or extranodal) | MALE | -22471 | ASIAN | 0 | 61 | 2012 | 7thStage IVNOYES | Dead |
| 13 | Diffuse large B-cell lymphoma (DLBCL) NOS (any anatomic site nodal or extranodal) | MALE | -13708 | WHITE | 0 | 37 | 2011 | 7thStage INONO | Alive |
| 14 | Diffuse large B-cell lymphoma (DLBCL) NOS (any anatomic site nodal or extranodal) | FEMALE | -21005 | ASIAN | 0 | 57 | 2010 | 7thStage IINONO | Alive |
| 15 | Diffuse large B-cell lymphoma (DLBCL) NOS (any anatomic site nodal or extranodal) | FEMALE | -19838 | ASIAN | 0 | 54 | 2010 | Stage INONO | Alive |
| 16 | Diffuse large B-cell lymphoma (DLBCL) NOS (any anatomic site nodal or extranodal) | MALE | -26520 | ASIAN | 0 | 72 | 2012 | 7thStage IINOYES | Alive |
| 17 | Diffuse large B-cell lymphoma (DLBCL) NOS (any anatomic site nodal or extranodal) | FEMALE | -30256 | WHITE | 0 | 82 | 2005 | 6thStage INONO | Alive |
| 18 | Diffuse large B-cell lymphoma (DLBCL) NOS (any anatomic site nodal or extranodal) | FEMALE | -13996 | ASIAN | 0 | 38 | 2011 | 7thStage IINONO | Alive |
| 19 | Diffuse large B-cell lymphoma (DLBCL) NOS (any anatomic site nodal or extranodal) | FEMALE | -25292 | WHITE | 0 | 69 | 2012 | 7thStage IINONO | Alive |
| 20 | Diffuse large B-cell lymphoma (DLBCL) NOS (any anatomic site nodal or extranodal) | FEMALE | -17023 | WHITE | 0 | 46 | 2010 | Stage IVYESYES | Alive |
| 21 | Diffuse large B-cell lymphoma (DLBCL) NOS (any anatomic site nodal or extranodal) | FEMALE | -21330 | ASIAN | 0 | 58 | 2013 | 7thNONO | Alive |
| 22 | Diffuse large B-cell lymphoma (DLBCL) NOS (any anatomic site nodal or extranodal) | FEMALE | -21021 | WHITE | 0 | 57 | 2000 | 5thStage IVNONO | Dead |
| 23 | Diffuse large B-cell lymphoma (DLBCL) NOS (any anatomic site nodal or extranodal) | FEMALE | -28474 | WHITE | 0 | 77 | 2005 | 6thStage IIIYESNO | Alive |
| 24 | Diffuse large B-cell lymphoma (DLBCL) NOS (any anatomic site nodal or extranodal) | MALE | -16720 | WHITE | 0 | 45 | 2004 | 6thStage IINONO | Alive |
| 25 | Diffuse large B-cell lymphoma (DLBCL) NOS (any anatomic site nodal or extranodal) | MALE | -25256 | WHITE | 0 | 69 | 2008 | 6thStage IVYESNO | Alive |
| 26 | Diffuse large B-cell lymphoma (DLBCL) NOS (any anatomic site nodal or extranodal) | MALE | -27255 | ASIAN | 0 | 74 | 2010 | 7thStage IVYESYES | Alive |
