# Supplementary material for: Expression of Tryptophan Metabolism Enzymes in Patients with Diffuse Large B‐cell Lymphoma and NK/T‐cell Lymphoma
Source: Cancer Med. 2023 May 6;12(11):12139–48. doi: 10.1002/cam4.5903 (PMC10278463; doi:10.1002/cam4.5903)
Supplement: Supplementary file 2 — Table S2. [file CAM4-12-12139-s002.docx]

**Table S2. Clinical characteristics of patients**

|  | **DLBCL**  **(n=43)** | **NK/TCL**  **(n=23)** |
| --- | --- | --- |
| **Gender** |  |  |
| Male | 20 (46.5%) | 19 (82.6%) |
| Female | 23 (53.5%) | 4 (17.4%) |
| **Age (year)** | 56.5±13.3 | 43±16.4 |
| **Advanced Lugano stage†** |  |  |
| Ⅰ | 3 (8.6%) | 4 (25.0%) |
| Ⅱ | 5 (14.3%) | 5 (31.3%) |
| Ⅲ | 5 (14.3%) | 0 (0) |
| Ⅳ | 22 (62.8%) | 7 (43.7%) |
| **LDH (U/L) †** | 282.6±83.9 | 364.5±292.8 |

DLBCL: diffuse large B-cell lymphoma; NK/TCL: natural killer/T-cell lymphoma.

Values are number of patients (percentage) or mean value ± standard deviation.

† Some cases were not taken into calculation due to loss of clinical data.
